# An RNA decay factor wears a new coat: UPF3B modulates translation termination

**DOI:** 10.12688/f1000research.12704.1

**Published:** 2017-12-20

**Authors:** Zhaofeng Gao, Miles Wilkinson

**Affiliations:** 1 Department of Reproductive Medicine, University of California San Diego Medical Center, La Jolla, CA, USA

**Keywords:** Nonsense-mediated RNA decay, RNA, UPF3B, translation termination

## Abstract

Nonsense-mediated RNA decay (NMD) is a highly conserved and selective RNA turnover pathway that has been subject to intense scrutiny. NMD identifies and degrades subsets of normal RNAs, as well as abnormal mRNAs containing premature termination codons. A core factor in this pathway—UPF3B—is an adaptor protein that serves as an NMD amplifier and an NMD branch-specific factor. UPF3B is encoded by an X-linked gene that when mutated causes intellectual disability and is associated with neurodevelopmental disorders, including schizophrenia and autism. Neu-Yilik *et al*. now report a new function for UPF3B: it modulates translation termination. Using a fully reconstituted *in vitro* translation system, they find that UPF3B has two roles in translation termination. First, UPF3B delays translation termination under conditions that mimic premature translation termination. This could drive more efficient RNA decay by allowing more time for the formation of RNA decay-stimulating complexes. Second, UPF3B promotes the dissociation of post-termination ribosomal complexes that lack nascent peptide. This implies that UPF3B could promote ribosome recycling. Importantly, the authors found that UPF3B directly interacts with both RNA and the factors that recognize stop codons—eukaryotic release factors (eRFs)—suggesting that UPF3B serves as a direct regulator of translation termination. In contrast, a NMD factor previously thought to have a central regulatory role in translation termination—the RNA helicase UPF1—was found to indirectly interact with eRFs and appears to act exclusively in post-translation termination events, such as RNA decay, at least *in vitro*. The finding that an RNA decay-promoting factor, UFP3B, modulates translation termination has many implications. For example, the ability of UPF3B to influence the development and function of the central nervous system may be not only through its ability to degrade specific RNAs but also through its impact on translation termination and subsequent events, such as ribosome recycling.

## Introduction

Gene expression is typically thought to be controlled at the level of RNA synthesis (transcription), but equally critical for determining steady-state RNA levels is the rate of RNA turnover. Among the best-characterized eukaryotic RNA turnover mechanisms is nonsense-mediated RNA decay (NMD), a highly conserved and selective pathway that detects and degrades mRNAs with translation stop codons positioned in specific (often suboptimal) contexts ^1– 5^. Originally regarded as a quality-control mechanism that rapidly degrades aberrant mRNAs with premature termination codons (PTCs), it is now clear that NMD also degrades many normal RNAs ^5^. Just as transcription factors regulate the rate of RNA synthesis from subsets of normal genes, NMD regulates the stability of subsets of normal mRNAs. While the physiological significance of this is not certain, increasing evidence strongly suggests that NMD is a highly regulated pathway that degrades subsets of mRNAs to influence specific biological events, including during development and in response to stress ^3, 5^.

**Figure 1.**
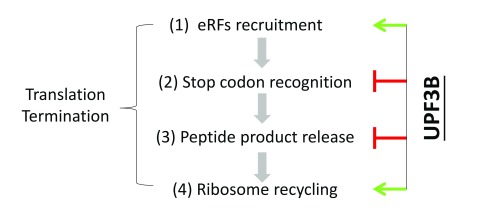
UPF3B regulates translation termination. In eukaryotes, translation termination proceeds through four consecutive stages. Using a reconstituted *in vitro* translation termination system, Neu-Yilik *et al*. ^11^ provided evidence that UPF3B modulates all four of these stages. They obtained evidence that UPF3B delays the middle stages, which may serve to regulate translation termination in RNAs with stop codons in ‘premature’ contexts. By contrast, UPF3B accelerates ribosome recycling, which may serve many functions, including facilitating the exposure of the mRNA body to nuclease attack during NMD. Of note, steps 2 and 3 are difficult to distinguish and thus it is possible that UPF3B acts on only one of these two steps. eRF, eukaryote release factor.

**Figure 2.**
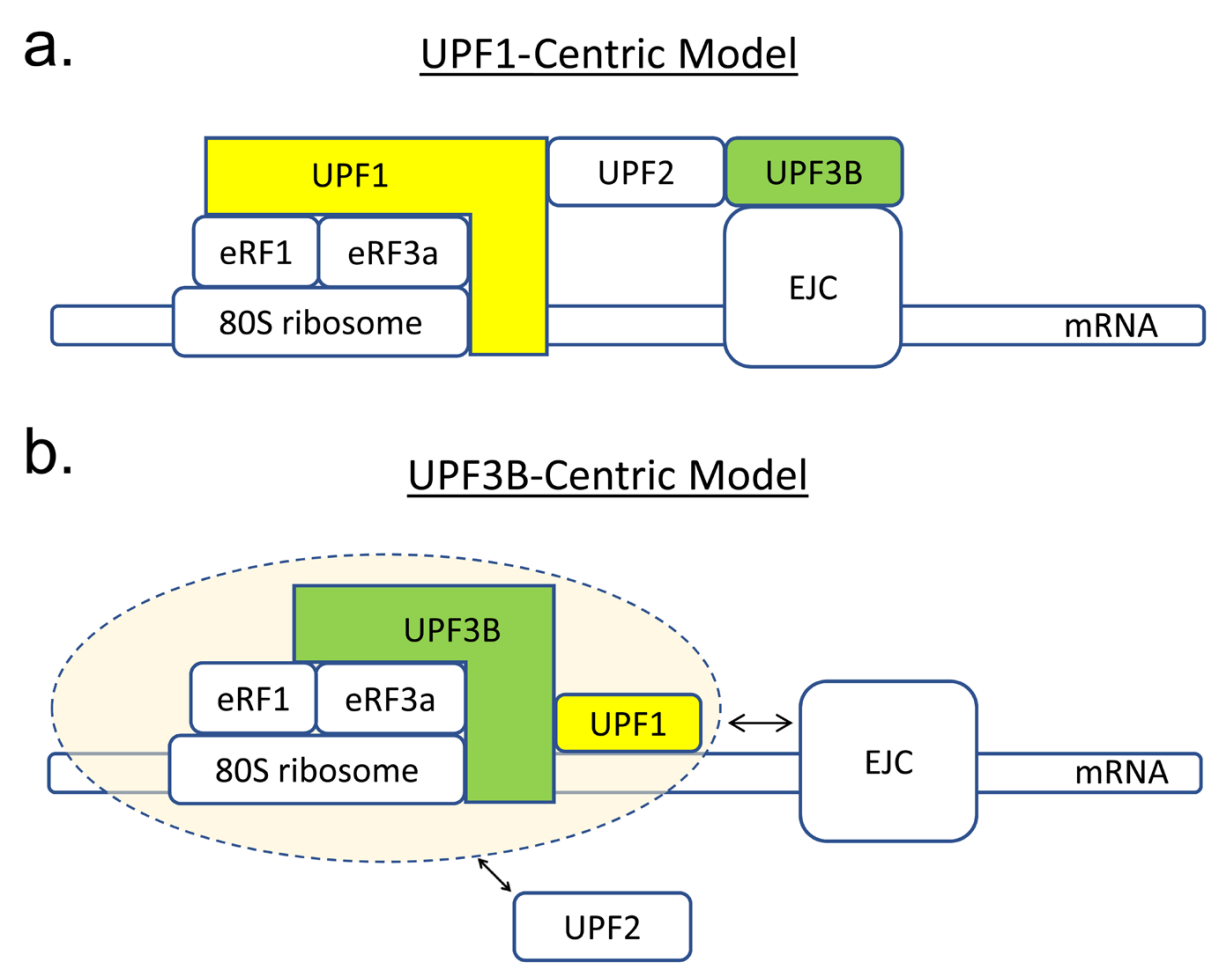
Two models that explain coupling of translation termination and nonsense-mediated RNA decay (NMD). ( **a**) In one model, UPF1 is the major factor molecularly connecting translation termination with NMD ^1, 22^. UPF1 interacts (directly or indirectly) with release factors (eRF1 and eRF3A) bound to the terminating ribosome stalled at a premature stop codon (PTC), based on co-immunoprecipitation analysis. UPF1 then recruits the adaptor protein, UPF2, which, in turn, binds to UPF3B, which is recruited to RNA by the exon junction complex (EJC) bound at splice junctions. UPF2 induces a conformational change in UPF1, which activates it for subsequent events that lead to RNA decay. ( **b**) In the UPF3B-centric model from Neu-Yilik *et al*. ^11^, UPF3B serves as an active assembly mediator that brings the translation termination machinery and the NMD machinery together (pink oval). Neu-Yilik *et al*. ^11^ found that UPF3B directly binds to RNA and interacts with ribosomes, which, according to their model, allows it to be loaded directly onto translated RNAs. At a PTC, UPF3B recruits the eRF1-eRF3-GTP complex, which leads to delayed translation termination. Neu-Yilik *et al*. ^11^ also demonstrated that UPF3B directly binds to UPF1 (without the necessity for UPF2 to serve as an adaptor), providing a mechanism by which UPF2-independent NMD occurs ^28^. It remains for future studies to determine how UPF2 and the EJC functionally interact (double-head arrows) with the NMD and translation termination machineries.

Many proteins have been identified that function in NMD, including those that act in specific branches of the NMD pathway ^2^. One of these branch-specific factors is UPF3B, which has been shown to promote the decay of specific mRNAs in a cell type-specific manner ^6– 10^. In a recent article in the EMBO Journal, Neu-Yilik *et al*. ^11^ report that UPF3B has a new role. They demonstrate that UPF3B modulates specific steps in translation termination ( Figure 1). Remarkably, they provide evidence that UPF3B has a central role in translation termination, as it interacts with ribosomes, it binds to RNA, and it directly interacts with release factors (RFs), the proteins that recognize stop codons in order to trigger translation termination ( Figure 2 and Supplemental Figure 1). These discoveries significantly alter the view we have of UPF3B and they have implications for how we view the multiple steps of gene expression. The authors’ findings deepen our understanding of the underlying mechanism of translation termination and have potential consequences in terms of biology and neural disease, as we detail below.

### NMD

NMD is a translation-dependent pathway triggered by in-frame stop codons in specific contexts. The core components of the NMD pathway—UPF1, UPF2, and UPF3 ( Figure 2)—are conserved in all known eukaryotes. UPF1 is an RNA helicase that processively remodels RNA and ribonucleoproteins (RNPs) ^12^. UPF2 serves as an adaptor protein that bridges UPF1 with UPF3 ^1^. In vertebrates, there are two UPF3 proteins with different functions: UPF3B is an NMD activator encoded by an X-linked gene that promotes a specific branch of the NMD pathway ^2, 6, 13– 15^, while UPF3A is a weak NMD factor encoded by an autosomal gene that recently was shown to be predominantly an NMD repressor in many cell types ^14, 16, 17^. In mammals, UPF3B interact with the exon junction complex (EJC), a set of proteins deposited just upstream of exon-exon junctions after RNA splicing ^13^. The EJC is thought to be responsible for the fact that in-frame stop codons more than about 50 nucleotides upstream of the last exon-exon junction almost always trigger NMD. The likely mechanism responsible for this “-50 boundary rule” is that EJCs bound at exon-exon junctions more than about 50 nucleotides downstream of the stop codon are not displaced by ribosomes (given the footprint lengths of the EJC and ribosome) and thus such bound EJCs are able to recruit factors, including UPF3B, which elicit RNA decay through the NMD pathway ^3, 13^.

### Translation termination

The termination of translation in eukaryotes requires the release factors (that is, eukaryotic RF1 (eRF1) and eRF3). When a translating ribosome reaches a stop codon on an mRNA, the ribosome recruits eRF1 in a complex with the GTPase, eRF3, bound by GTP. eRF1 recognizes the stop codon by virtue of its mimicking the shape of tRNA. Stop codon recognition triggers eRF3 to hydrolyse GTP, leading to a conformational change of eRF1, peptidyl-tRNA hydrolysis, and dissociation of the nascent polypeptide and eRF3 from the ribosome. The essential ATP-binding cassette protein, ABCE1, then splits 80S ribosomes into free 60S subunits and 40S subunits ^18, 19^, followed by the release of the ribosomal subunits from the mRNA ^20^.

### The NMD factor, UPF3B, functions in translation termination

While normal translational termination is well understood, the mechanisms underlying premature translation termination remain relatively obscure. Prevailing models for the mechanics of premature translation termination ascribe a critical role for the NMD factor UPF1 ^21– 23^. In support, it has been found that genetic ablation of *UPF1* in yeast leads to increased stop codon suppression ^24, 25^ and RNA interference–mediated depletion of UPF1 in human cells reduces stop codon read-through ^26^. In support of a direct role, UPF1 was found to interact with both eRF1 and eRF3A, based on co-immunoprecipitation (co-IP) experiments ( Figure 2a) ^25– 27^. These and other findings support a model in which UPF1 recruits eRFs to ribosomes that are stalled at a premature termination codon in an early phase of termination and then UPF1 promotes ribosome disassembly in a late phase of termination via its ATPase function, activated by UPF2 and UPF3 binding ^21, 22, 29^ ( Figure 2a).

While an attractive model, it has not been possible to experimentally test this model because there is no reliable *in vivo* termination assay. Thus, to test the validity of this model, Neu-Yilik *et al*. ^11^ chose instead to use a fully reconstituted *in vitro* mammalian system that recapitulates all phases of translation termination ^30– 32^. Using this *in vitro* system, these investigators found that neither UPF1 nor UPF1’s biochemical functions (such as ATP binding or hydrolysis), nor its phosphorylation, plays a discernible role in early or late phases of translation termination. Furthermore, they found that UPF1 does not measurably bind eRF1 and eRF3A directly, and thus the previously described interactions between UPF1 and eRFs (identified in co-IP experiments) are likely to be indirect. The data from Neu-Yilik *et al*. ^11^ suggest that UPF1 is functionally dispensable during translation termination *in vitro* and that the essential role of UPF1 (as well as UPF2) is exerted exclusively in the post-termination phase of translation, such as the rapid RNA decay elicited by NMD. Intriguingly, Neu-Yilik *et al*. ^11^ obtained evidence that another NMD factor—UPF3B—is instead critical for translation termination. Under conditions simulating premature translation termination, UPF3B delays termination and inhibits peptide release. UPF3B also has a second function: it promotes dissociation of post-termination ribosomal complexes after release of the nascent peptide. As we discuss below, the discovery of these new UPF3B functions alters how we view this NMD factor and lends further support to the growing view that translation termination and RNA decay are coupled.

## UPF3B modulates translation termination

Translation termination at a PTC is thought to be less efficient than when it occurs at a normal stop codon. To examine the role of the UPFs in this process, Neu-Yilik *et al*. ^11^ used a fully reconstituted translation termination system that had previously been shown to mimic translation termination ^33^. In this system, ribosome subunits, amioacylated tRNAs, and a complete set of purified individual translational factors are assembled on a model mRNA encoding a tetrapeptide followed by a UAA stop codon. Neu-Yilik *et al*. ^11^ introduced various combinations of purified NMD factors to test their roles in this system. To simulate the inefficient translation termination at PTCs, the authors used limiting amounts of RFs.

Under this set of conditions, the authors found that introduction of UPF3B delayed translation termination, as assayed by a toeprinting assay. This assay showed that UPF3B dramatically reduced the ability of pre-termination complexes (preTCs) to convert into post-termination complexes (postTCs). A UPF3B mutant lacking the EJC-interaction domain that is defective in NMD was fully competent in inhibiting translation termination. This is an important finding from a mechanistic perspective and also has practical applications. For example, this mutant may allow one to tease out the relative role of UPF3B’s NMD activity from its UPF3B’s translation termination activity in different biological scenarios.

Interestingly, UPF2, which had no effect on its own, reversed the inhibitory role of UPF3B. Thus, UPF3B and UPF2 appear to act in a ‘yin-yang manner’ to modulate translation termination, perhaps for regulatory purposes.

To determine whether UPF3B downmodulates translation termination by inhibiting peptidyl-tRNA hydrolysis, Neu-Yilik *et al*. ^11^ tracked the progress of peptide release by using ^35^S-labeled initiator-tRNA in a reconstituted termination reaction. They found that the addition of UPF3B reduced the efficiency of peptidyl-tRNA hydrolysis under the same reaction conditions in which UPF3B inhibited translation termination. These data are consistent with UPF3B inhibiting translation termination by weakening peptidyl-tRNA hydrolysis.

A potential caveat with the above experiments is that the authors used excess amounts of UPF3B and other NMD proteins to saturate their interactions with other components of the translation termination machinery. Although this saturating approach successfully illuminated new functions for UPF3B, it will be important, in the future, to perform dose-response experiments to determine the functions of UPF3B when present at limiting concentrations.

## UPF3B forms a complex with release factors

To assess whether UPF3B might function directly in translation termination, Neu-Yilik *et al.* ^11^ examined whether UPF3B forms complexes with RFs *in vivo*. Through co-IP experiments (in the presence of RNases to ablate RNA-dependent interactions), they found that UPF3B interacts with eRF3A ( Figure 2b and Supplemental Figure 1a). In contrast, UPF3B did not measurably interact with eRF1 ( Supplemental Figure 1a). However, UPF3B inhibited the ability of UPF1 to interact with eRF1, providing evidence that UPF3B does have an active role in eRF1 complexes. This negative regulatory role is consistent with UPF3B’s ability to inhibit translation.

To elucidate whether UPF3B directly interacts with eRFs, Neu-Yilik *et al*. ^11^ performed *in vitro* pull-down experiments. This revealed that UPF3B directly binds with both eRF1 and eRF3A ( Figure 2b and Supplemental Figure 1a, 1b). Surprisingly, this assay showed that other core NMD factors, including UPF1, did not directly interact with eRFs. This goes against the widely held assumption that UPF1 has a direct and integral role in translation termination (see the Introduction and below). Together, these data raised the possibility that, instead, UPF3B is the NMD factor that plays the most direct role in regulating translation termination.

Follow-up experiments using size exclusion chromatography (SEC) under physiological buffer conditions provided evidence that UPF3B forms a stable trimeric complex with both eRF1 and eRF3A ( Figure 2b and Supplemental Figure 1a, 1b). In experiments in which UPF3B was mixed with eRF1 and eRF3A individually, UPF3B was found to bind strongly with eRF3A but only weakly (probably transiently) with eRF1 ( Supplemental Figure 1a), the latter of which is consistent with the lack of measurable interaction of UPF3B with eRF1, as measured by co-IP ( Supplemental Figure 1a). SEC analysis also provided evidence that the stoichiometric relationship of eRF1, eRF3A, and UPF3B is about 1:1:1. Both *in vitro* pull-down and SEC assays demonstrated that this trimeric complex is stable *in vitro* ( Figure 2b and Supplemental Figure 1b) and its formation is independent of the eRF3A substrate GTP. However, this does not rule out that GTP hydrolysis by eRF3A could modulate the interactions between itself and eRF1 and UPF3B, an interesting question to address in the future.

**Figure 3.**
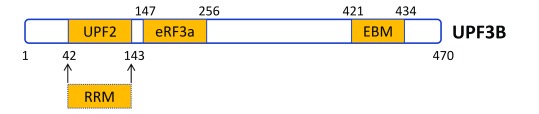
The interaction sites on human UPF3B for its cofactors. The numbers denote the positions of amino acids in human UPF3B. The RNA recognition motif (RRM)-like domain overlaps with the UPF2-interacting domains ^35^. While Neu-Yilik *et al*. ^11^ established that UPF3B directly binds to RNA, whether the UPF3B RRM domain is involved is not known. EBM, exon junction complex binding motif.

Neu-Yilik *et al*. ^11^ then went on to perform mutational analysis to understand how UPF3B interacts with eRF3A. They found that UPF3B likely interacts with the amino (N)-terminus of eRF3A, based on the inability of an N-terminal eRF3A mutant to interact with UPF3B, both *in vitro* and *in vivo*. This is consistent with a regulatory role for UPF3B, as eRF3A does not require the N-terminus for its function in translation termination ^34^. Using both *in vitro* pull-down and co-IP analyses, they showed that eRF3A binds to UPF3B in a middle domain between UPF3B’s UPF2- and EJC-interaction domains ( Figure 3). Thus, this study defined a new functional domain in UPF3B. The finding that UPF3B uses different binding sites for eRF3A, UPF2, and the EJC suggests that the interactions between UPF3B and these factors are not mutually exclusive ( Figure 2b).

## UPF3B triggers disassembly of post-termination complexes

To address the role of UPF3B in normal translation termination, Neu-Yilik *et al*. ^11^ performed experiments with saturating amounts of eRFs. Under this condition, they found that UPF3B not only delayed translation termination but destabilized the postTCs; that is, it triggered their release from the mRNA. This activity was independent of UPF1 and prevented by UPF2. Analysis of a battery of UPF3B mutants showed that UPF3B’s EJC-interaction domain was dispensable, and that neither the UPF2-interaction domain nor the eRF3A-interaction (middle) domain was sufficient for this function. Subsequent experiments indicated that UPF3B dissociates postTCs after both GTP and peptidyl-tRNA hydrolysis. In contrast, UPF3B did not measurably impact either preTCs or postTCs before peptide hydrolysis. UPF3B was found to dissociate postTCs regardless of whether they are bound by eRF3A, implying that UPF3B interactions with eRF3A are dispensable for UPF3B to promote the dissociation of the ribosome from the mRNA.

## Model

To understand how UPF3B might regulate translation termination, Neu-Yilik *et al*. ^11^ examined whether it might interact with ribosomes. Using a co-sedimentation assay, they found that UPF3B shares with UPF1 the ability to associate with ribosomes ( Figure 2b and Supplemental Figure 1a, 1b). In addition, they found that UPF3B interacts directly with RNA *in vitro* ( Figure 2b and Supplemental Figure 1a), consistent with the RNA-binding activity of UPF3B previously identified by individual-nucleotide resolution cross-linking and immunoprecipitation (iCLIP) analysis ^36^. These findings raise the exciting possibility that UPF3B can independently bind the ribosome and the mRNA without requiring additional bridging by other NMD proteins or the EJC. Thus, like UPF1, UPF3B is able to directly load onto actively translated RNAs, which has important implications for devising models explaining how UPF3B acts in translation termination and NMD.

An outstanding question is how UPF3B binds to RNA. UPF3B was previously shown to have a domain strongly reminiscent of an RNA-recognition motif (RRM), but this was shown to be responsible for binding to UPF2, not RNA ^35^.

The findings from Neu-Yilik *et al*. ^11^ led the authors to propose a new model in which UPF3B has a central role in translation termination and NMD ( Figure 1). In this model, UPF3B binds in the vicinity of the A site of the ribosome and assists in the recruitment of eRF1-eRF3A during the initial slow phase of termination at a PTC ( Figure 1, phase 1). UPF3B then delays termination by sterically impeding stop codon recognition and peptide release by eRF1 ( Figure 1, phase 2). After a retarded phase of peptide release ( Figure 1, phase 3), UPF3B contributes to the rescue of ribosomes stalled at a PTC by promoting the release of postTCs ( Figure 1, phase 4). According to their model, this last phase is independent of eRF binding and is promoted by interactions of UPF3B with the ribosome subunit interface. Subsequently, UPF1, UPF2, and UPF3B drive 3′ untranslated region (UTR) remodeling and the recruitment of enzymes responsible for the decay phase of NMD.

## UPF1 does not measurably impact translation termination

The work of Neu-Yilik *et al*. ^11^ is important not only because of the new roles it defines for UPF3B but also for the insights it provides into the role of UPF1. As described in the Introduction, several previous lines of evidence had suggested that this central NMD factor has direct roles in translation termination. Thus, it was a surprise when Neu-Yilik *et al*. ^11^ found that recombinant UPF1 had no measurable effect on two steps regulated by UPF3B: (i) the preTC-to-postTC transition and (ii) release of the postTC from the mRNA. Furthermore, UPF1 failed to have a measurable effect regardless of whether there were limiting or saturating amounts of eRFs. While the authors confirmed previous reports ^26, 27, 37^ that UPF1 interacts with eRFs (through co-IP experiments), they found, using an *in vitro* pull-down assay, that UPF1 does not measurably directly bind with either eRF1 or eRF3A ( Supplemental Figure 1a). Thus, UPF1 interacts indirectly with eRFs. At first glance, the most obvious mechanism for this indirect binding is through the adaptor protein UPF2, as UPF1 directly binds to UPF2, and UPF2 directly binds to the eRF-binding protein UPF3B ^13^. However, this seems unlikely, as Neu-Yilik *et al.* ^11^ found that UPF2 did not measurably interact with eRF1 and only minimally interacted with eRF3A ( Supplemental Figure 1a). This raised the possibility that instead UPF1 directly binds to UPF3B, which they tested by the pull-down assay and found to be the case ( Figure 2b and Supplemental Figure 1a). Based on SEC analysis, UPF1 and UPF3B bound to each other with a stoichiometry of about 1:1. Of note, an earlier study concluded that UPF3B does not directly interact with UPF1 ^38^, but this study used a truncated UPF1 variant.

Because ATP binding and hydrolysis are essential for UPF1’s role in NMD ^1^, Neu-Yilik *et al*. ^11^ also examined whether these biochemical steps impact translation termination. They found that translation termination *in vitro* is independent of both ATP binding and UPF1 ATP hydrolysis, as shown in toeprinting experiments by adding ATP or its non-hydrolyzable analog AMPPNP. Neu-Yilik *et al*. ^11^ also investigated the role of UPF1 phosphorylation, as this post-translation modification is considered an integral step in NMD ^39– 41^. According to current models, UPF1 phosphorylation occurs through formation of a SMG1-UPF1-eRF1-eRF3 (SURF) complex composed of UPF1, eRF1, eRF3, the UPF1 kinase SMG1, and two NMD factors (SMG8 and SMG9) known promote UPF1 phosphorylation ^13^. This SURF complex is thought to assemble at the site of translation termination and phosphorylate UPF1, leading to the recruitment of RNA decay-promoting factors ^1^. To test the role of UPF factors on SURF function, Neu-Yilik *et al*. ^11^ examined the effect of SMG1/8/9 on translation termination, in the presence or absence of UPF1, but found no measurable effect. The authors also examined UPF1 phosphorylation and found that UPF3B modestly inhibited the addition of this post-translational modification. In contrast, when UPF3B and UPF2 were added together, UPF1 phosphorylation was strongly inhibited. It is tempting to speculate that the repression of UPF1 phosphorylation by the other UPFs influences post-translation termination mechanisms such as NMD.

Collectively, the results from Neu-Yilik *et al*. ^11^ raise the possibility that UPF1 is dispensable for translation termination, and hence the function of UPF1 may be exclusively for post-termination events, such as the decay of transcripts through the NMD pathway. This notion is consistent with previous studies showing that UPF1 phosphorylation and its ATPase and helicase activities are important for UPF1’s functions in 3′-UTR mRNP remodeling and the recruitment of mRNA decay factors in metazoans ^12, 42– 44^.

A potential caveat with the experiments performed by Neu-Yilik *et al*. ^11^ is that even though limiting amounts of RFs were used to mimic premature termination, translation termination was relatively efficient, as indicated by the toeprinting assay. Thus, the conditions used by the authors could have masked a stimulatory effect of UPF1 on translation termination. Indeed, this possibility is supported by recent experiments in *Saccharomyces cerevisiae* showing that Upf1p promote translation termination and ribosome release at PTCs ^45^. While it remains to be determined if yeast and mammalian UPF1 differ in their ability to stimulate translation termination, it would not be surprising if they do differ, as the mammalian NMD pathway appears to be more complex than the NMD pathway in lower organisms. Mammalian NMD requires a large number of factors, appears to have several branches, and is controlled by diverse regulatory mechanisms, whereas yeast NMD appears to require fewer factors and is a single linear pathway.

## Perspective

### Physiological implications of UPF3B’s ability to regulate translation termination

The finding by Neu-Yilik *et al*. ^11^ that UPF3B is not only an RNA decay-promoting factor but influences translation termination was unexpected. What might be the physiological relevance of this? For example, what benefit would be provided by UPF3B’s ability to delay translation termination? One possibility is that this activity serves to stimulate the decay of RNAs targeted by the NMD pathway. Delayed translation termination would likely increase the time required to assemble NMD-promoting complexes, thereby increasing the probability that such an RNA would be degraded by NMD. In addition, paused ribosomes would be predicted to lead to reduced translation of ribosomes upstream, which could leave the RNA more exposed to nucleases. Support for this notion comes from *S. cerevisiae*, where it has been shown that slower translation elongation rates reduce translation initiation, which, in turn, stimulates mRNA decapping and degradation ^46– 48^.

What might be the physiological relevance of UPF3B stimulating the release of postTCs? The most obvious benefit conferred by this activity would be an enhancement of ribosome recycling. This would not only be energetically favorable for cells, but it would likely increase the efficiency of translation as well as translation-dependent activities, including NMD. Another possible benefit of the UPF3B-dependent quick release of postTCs stalled at PTCs is facilitation of mRNA exposure to ribonucleases for complete degradation. Indeed, recent studies in yeast support the notion that postTC release promotes RNA decay ^45^.

The finding that UPF3B regulates translation termination is intriguing in light of the fact that all known vertebrates harbor a second copy of the *UPF3* gene. Might this other *UPF3* paralog— *UPF3A*—encode a protein that also functions in translation termination? One possibility is that UPF3A promotes UPF3B’s translation termination functions. This follows from two findings: (i) the ability of both UPF3A and UPF3B to directly bind to the NMD factor, UPF2, and (ii) the discovery by Neu-Yilik *et al*. ^11^ that UPF2 inhibits UPF3B’s translation termination functions. By binding and thereby presumably sequestering UPF2, UPF3A could relieve UPF2’s inhibitory role and thus promote UPF3B-mediated effects on translation termination.

### Does UPF3B influence translation termination in cells?

Neu-Yilik *et al*. ^11^ provide strong evidence that UPF3B influences premature translation termination *in vitro.* Does it also have this activity in live cells? Consistent with this possibility is the evidence that ribosome association is prolonged at premature stop codons in *S. cerevisiae* and mammalian cells ^49, 50^. Indeed, some evidence suggests that the kinetics of termination discriminates between normal termination codons and PTCs *in vivo* ^51, 52^. The ability of UPF3B to promote the disassembly of post-termination complexes is consistent with the finding that deletion of *UPF3*, as well as the other *UPF* genes, causes defects in ribosome release in yeast, both *in vitro* and *in vivo* ^53^.

To more directly test whether UPF3B functions in translation termination in cells, one could use ribosome profiling (Riboseq), a powerful technique that monitors translation genome-wide *in vivo* by deep sequencing of ribosome-protected mRNA fragments ^54– 56^. Riboseq has been successfully applied to analyze translation termination defects and ribosome recycling defects in many different contexts ^54^. If UPF3B promotes ribosome recycling, this predicts that cells from *Upf3b*-null mice ^57^ would show increased ribosomes stalling or queuing upstream of in-frame stop codons. This Riboseq analysis would also distinguish between UPF3B acting as a general ribosome release factor vs. a selective ribosome release factor targeting only specific RNAs, such as those ultimately degraded by NMD.

### Potential implications of the new functional domain in UPF3B

Another important finding of Neu-Yilik *et al*. ^11^ is their identification of a new functional domain in UPF3B. They found that its middle region, which previously had no known function, serves as a direct binding site for eRF3A ( Figure 3). Interestingly, mutations in this particular region of *UPF3B* are found in patients with intellectual disability (ID); indeed, mutations in this region have been shown—through human pedigree analysis—to likely cause ID and even some neuro-developmental disorders such as autism spectrum disorder (ASD) and schizophrenia (SCZ) ^58– 60^. This region of UPF3B appears to be critical for mature neuron functions, based on a study by Alrahbeni *et al*. ^61^. These authors investigated the functional effect of missense mutations in this region from several different male patients with ID, some of whom also displayed ASD or SCZ. Using a gain-of-function tethering assay, the authors found that the mutant UPF3B proteins produced as a result of these missense mutations caused dramatically reduced NMD activity in both neural and non-neural cell lines. To test whether these mutant proteins influenced neuronal maturation, they transiently overexpressed them in neural cells cultured under differentiation conditions. They found that these mutant UPF3B proteins dramatically decreased neurite arborization, the process by which neurons generate branch-like dendritic processes in order to enhance neural connectivity with neighboring cells. This arborization defect was specific, as overexpression of wild-type UPF3B had no significant effect on arborization. The discovery that specific *UPF3B* mutations lead to reduced neurite arborization is significant, as both mouse models and post-mortem human studies have linked neuronal branching defects to neurodevelopmental disorders ^62– 64^.

These results from Alrahbeni *et al*. support a model in which UPF3B’s NMD function is critical for neuron arborization and consequent neural processes required for normal intellect and behavior. However, might UPF3B’s role in translation termination be responsible instead? Such an alternative model predicts that human ID patients with *UPF3B* missense mutations in the middle domain will express a form of UPF3B unable to bind to eRF3A and unable to regulate translation termination. If this model proves to be correct, it could potentially resolve a paradoxical finding made by another group—Jolly *et al*. ^65^. Studying post-mitotic hippocampal neurons, Jolly *et al*. found that short hairpin RNA-mediated depletion of UPF3B caused a significant increase in arborization of both axons and dendrites ^66^. Thus, the data of Jolly *et al*. support the notion that UPF3B *inhibits* arborization, whereas Alrahbeni *et al*. obtained evidence that UPF3B *promotes* arborization ^61, 65^. How can their opposite results be reconciled? One possibility is that UPF3B’s two functions—NMD and translation termination—exert opposite roles in arborization and are differentially affected by UPF3B depletion ^66^ and mutant UPF3B overexpression ^61^.

One means to dissect the biological roles of UPF3B’s different biochemical activities is to examine the effect of UPF3B mutants that lose one activity but retain another activity. In this regard, Neu-Yilik *et al*. ^11^ identified a UPF3B mutant (lacking the EJC-interaction domain) that has no NMD-promoting activity but is fully competent in inhibiting translation termination. By performing rescue experiments with this mutant versus wild-type UPF3B, one can begin to dissect the importance of UPF3B’s NMD versus UPF3B’s translation termination functions. By analogy, SMG6 mutants lacking either NMD function or telomerase maintenance function were used in rescue experiments to define the role of SMG6 in mouse embryonic stem cell differentiation ^63, 66^.

A simple prediction of the finding that UPF3B interacts with the N-terminus of eRF3A is that an N-terminal truncation protein of eRF3A would not be susceptible to UPF3B-mediated translation termination inhibition. Surprisingly, Neu-Yilik *et al*. ^11^ found that this was not the case. In addition, they found that the interaction between UPF3B and eRF3A interaction is dispensable for the ability of UPF3B to promote postTC dissociation. These results suggest that UPF3B modulates translation termination events independently of its ability to interact with eRF3A.

Poly-(A) binding protein (PABP) also binds the N-terminal region of eRF3A ^67, 68^. This is interesting given that PABP has the opposite functions as UPF3B, as PABP stimulates translation termination ^68^ and represses NMD ^69^. It is thus tempting to speculate that PABP and UPF3B compete for eRF3A binding as a means to determine whether an RNA is stabilized and translated or undergoes translation termination and is degraded. What mechanism might control this putative competition? One model is that the type of the stop codon determines the probability of whether eRF3A binds PABP or UPF3B. In this model, binding of PABP to eRF3A might be favored at a normal stop codon, leading to ribosome recycling, RNA stabilization, and further rounds of translation. In contrast, binding of UPF3B to eRF3A might be favored at a PTC, leading to delayed translation termination, activation of NMD, and consequent RNA decay.

### Elucidating the molecular choreography guiding how UPF factors interact with RFs

To understand the molecular mechanisms by which UPF3B collaborates with eRFs and other UPF proteins in translation termination, it will be critical to solve the crystal structures of UPF3B-containing complexes. Neu-Yilik *et al*. ^11^ obtained *in vitro* evidence for two stable UPF3B-containing complexes—UPF3B/eRF3A/eRF1 and UPF3B/eRF3A/UPF1—both of whose structures will be intriguing to decipher. The structure of the UPF3B/eRF3A/eRF1 complex would likely represent a snapshot of early events in translation termination whereas the UPF3B/eRF3A/UPF1 complex would likely represent later events, such as the transition between translation termination and NMD. Solving such structures would not only reveal how UPF3B and eRF3A interact, but they might suggest how eRF1 and UPF1 function in these complexes. Ultimately, structural analysis could help decipher how translation termination and NMD are coordinated.

There are many other intriguing questions regarding the role of UPF3B in translation termination. For instance, among its various binding partners, which one does UPF3B interact with first? Among the many biochemical steps that UPF3B could theoretically act in, which pathway does UPF3B preferentially take? Which reaction steps are rate-limiting and thus dictate the magnitude of UPF3B-mediated effects on translation termination? Does UPF3B reduce the affinity of eRFs for the terminating ribosome to inhibit translation termination? Or does UPF3B repress the ability of eRF3A to undergo GTP binding or hydrolysis? To address these questions, one could employ the strategy recently used to study factors involved in translation initiation ^70^. In this study, thermodynamic and kinetic analyses were performed on five essential single components (three RNA-binding proteins, RNA and ATP), coupled with computational simulation, to yield a comprehensive view of the molecular choreography driving translation initiation. To study the role of UPF3B in translation termination, a similar analysis could be done with all the components involved, including the three UPF factors, the two eRFs, the ribosome, RNA, and relevant small molecules (ATP and ADP bound by UPF1 and GTP and GDP bound by eRF3A). Elucidation of the molecular choreography by which UPF3B regulates translation termination could also serve as a model to understand other UPF-dependent post-transcriptional mechanisms, such as NMD itself.

## Coda

The unexpected discovery that a well-established factor with RNA decay-promoting activity has roles in translation leads to the question of which activity came first. Was UPF3B’s primordial role in NMD? Or did UPF3B first function in translation? Its primordial activity likely arose over 500 million years ago, as UPF3 is found throughout the phylogenetic scale, from yeast to humans. We know that UPF3 acts as an NMD factor in all species so far tested, but whether it has a role in translation termination remains to be determined for most species. Regardless of its evolutionary origin, the ability of UPF3B to function in both biosynthesis (translational regulation) and decay (RNA turnover) provides another example of how biology is driven by forces that simultaneously synthesize and degrade. In the words of T.S. Eliot: “April is the cruelest month, breeding Lilacs out of the dead land, mixing memory and desire, stirring dull roots with spring rain”.

## Abbreviations

ASD, autism spectrum disorder; co-IP, co-immunoprecipitation; EJC, exon junction complex; eRF, eukaryote release factor; ID, intellectual disability; NMD, nonsense-mediated RNA decay; N-terminus, amino terminus; PABP, poly-(A) binding protein; postTC, post-termination complex; preTC, pre-termination complex; PTC, premature termination codon; RF, release factor; Riboseq, ribosome profiling; RNP, ribonucleoprotein; SCZ, schizophrenia; SEC, size exclusion chromatography; SURF, SMG1–UPF1–eRF1–eRF3; UPF, up-frameshift; UTR, untranslated region.

## Supplementary material

**Supplemental Figure 1. Summary of the evidence for the interactions between eRF1, eRF3A, UPF3B, UPF2L, UPF1, mRNA, and the 80S ribosome, as reported in Neu-Yilik *et al*., 2017 ^11^**. (a) Two-component interactions detected by the indicated assays. ‘+’ and ‘−‘ denote interactions detected and not detected, respectively. The discrepancy between the *in vivo* co-immunoprecipitation (co-IP) and other *in vitro* assays likely reflects either indirect interactions meditated by other cofactors or through post-translational modifications. (b) Three-component complexes detected by the indicated assays. Blue denotes components in the examined complexes. PD, *in vitro* pull-down; SEC, size exclusion chromatography; SED, ribosome co-sedimentation.
